# Facility- and patient-level factors associated with implementation of contact precautions in hospitalized VA patients with positive CRE cultures

**DOI:** 10.1017/ash.2024.36

**Published:** 2024-05-03

**Authors:** Geneva M. Wilson, Margaret Fitzpatrick, Katie J. Suda, Linda Poggensee, Makoto Jones, Martin E. Evans, Charlesnika T. Evans

**Affiliations:** 1 Center of Innovation for Complex Chronic Healthcare (CINCCH), Hines Jr. Veterans Affairs Hospital, Hines, IL, USA; 2 Northwestern University Feinberg School of Medicine, Department of Preventive Medicine, Center for Health Services and Outcomes Research, Chicago, IL, USA; 3 Loyola Medical Center, Maywood, IL, USA; 4 Center for Health Equity Research and Promotion, VA Pittsburgh Heath Care System, Pittsburgh, PA, USA; 5 Department of Medicine, University of Pittsburgh School of Medicine, Pittsburgh, PA, USA; 6 Department of Veterans Affairs, VA Salt Lake City Healthcare System, Salt Lake City, UT, USA; 7 Department of Medicine, Division of Epidemiology, University of Utah, Salt Lake City, UT, USA; 8 Department of Veterans Affairs, Lexington VA Medical Center, Lexington, KY, USA

## Abstract

Decreasing the time to contact precautions (CP) is critical to carbapenem-resistant Enterobacterales (CRE) prevention. Identifying factors associated with delayed CP can decrease the spread from patients with CRE. In this study, a shorter length of stay was associated with being placed in CP within 3 days.

## Background

Carbapenem-resistant Enterobacterales (CRE) infections are considered an urgent threat by the Centers for Disease Control and Prevention ^1^with mortality rates reported as high as 50%.^2^ CRE causes healthcare-associated infections and outbreaks. Contact precautions (CP) are infection prevention measures that are usually enacted in response to the identification of CRE (and other critical organisms) in hospitalized patients.^3^ They often involve cohort isolation of infected patients away from uninfected patients, enhanced hand hygiene and protective personal equipment for healthcare workers interacting with infected patients, and heightened cleaning protocols for the patient room during admission and after discharge.^4^ The use of CP is instrumental in stopping the transmission of CRE between patients in healthcare settings. However, compliance with CP measures generally falls well short of 100%, and there is little evidence about factors associated with CP compliance, the identification of which could help optimize and target infection prevention strategies. The goal of this study was to determine factors associated with being placed in CP in a cohort of hospitalized patients with positive CRE cultures.

## Methods

A retrospective cohort study was conducted of hospitalized Department of Veterans Affairs (VA) patients with a positive CRE culture from all body sites between January 1, 2013, and December 31, 2018. Patients hospitalized at 127 VA Medical Centers across the United States and territories (Puerto Rico) were found and included.

### Data collection

The following information was collected from the Corporate Data Warehouse (CDW), the central repository for VA patient data: patient age, race/ethnicity, gender, facility complexity, facility region, and microbiology culture data. Admission date, CRE culture date, and date of death/discharge were also collected. For patients whose CP were identified via CDW data, the date of the earliest CP associated with the admission of interest was retrieved.

### Chart review

For 18% of patients included in the cohort, there was no order for CP identified in the CDW. A chart review of nursing orders, infectious disease consult notes, and daily progress notes were performed to confirm the presence/absence of CP for those admissions. Written evidence of CP in any of these records was considered evidence of CP, and the start date for CP was recorded as the first date of evidence in the medical record. If no written evidence of CP was found for the entire admission, then the patient was considered to not have had CP enacted. Besides confirmation of CP, the following information was also collected: number of days between admission and culture date, number of days between culture date and enaction of CP, and number of days between culture date and patient discharge or death date. The reason for admission, total length of stay, and specimen type were also collected.

### Outcome definition and statistical analysis

CP were defined at 2 time points: 3 days and 7 days post culture collection date. Comparisons were made between those with CP within the defined time ranges and those without. Data were analyzed using descriptive statistics. Categorical variables were analyzed using *χ* ^2^ and Fisher’s exact tests. Continuous variables were analyzed using Wilcoxon’s signed rank test. Significant variables were defined as those with a *P*-value of .05. Analysis was done using SAS 9.7.

## Results

During the study, 1,339 CRE inpatient cultures were identified, most of which (1,089) were extractable in the CDW. After removing duplicates and cultures for whom CP were enacted prior to the CRE cultures, 493 cultures from 449 unique patients remained from the CDW cohort. For 250 cultures, CP could not be found using CDW data. After duplicate removal, 199 cultures from 199 patients remained. After removing patients whose CP preceded the CRE culture of interest, 186 patients remained. The final cohort for this analysis contained 679 cultures from 635 unique patients. Included patients were predominantly male (97.5%), White (65.4%), and older (mean age = 72.2 ± 11.8 yr).

43.9% of patients were placed in CP within 3 days of their culture (Table 1). Patients placed in precautions within 3 days were older than those not placed within 3 days (mean age 74.5 yr, std (12.0) vs 70.3 (11.2), *P*-value < .0001). Patients in CP were also more likely to come from the highest level of facility complexity (79.2% vs 71.4%, *P*-value = .0056). Placement in CP within 3 days was also more likely to occur in facilities outside the continental United States (41.3%) and occurred less frequently in the South (34.9%). Those with CP had a median admission length of 11 days compared with 30 days for those without precautions (*P*-value < .0001). They also were cultured earlier in their admission compared to those without precautions (median 0.0 d vs 3.0 d, *P*-value < .0001).

**Table 1.** Factors associated with placement in contact precautions within 3 and 7 days of culture (N = 679)

	Contact precautions within 3 days (n = 298, 43.9%)	Contact precautions outside 3 days (n = 381, 56.2%)	*P*-value	Contact precautions within 7 days (433, 63.8%)	Contact precautions outside 7 days (246, 36.2%)	*P*-value
**Age (mean, std)**	74.5 (12.0)	70.3 (11.2)	**<.0001**	73.8 (11.7)	69.4 (11.4)	**<.0001**
**Race/ethnicity**			.2181			.2825
White	202 (67.8%)	241 (63.8%)		287 (66.3%)	156 (63.4%)	
Black	79 (26.5%)	124 (32.6%)		122 (28.2%)	81 (32.9%)	
Other /missing	17 (5.7%)	16 (4.2%)		24 (5.5%)	9 (3.7%)	
**Gender**			.4462			.9352
Male	289 (97.0%)	373 (97.9%)		422 (97.5%)	240 (97.6%)	
Female	9 (3.0%)	8 (2.1%)		9 (2.5%)	6 (2.4%)	
**Facility complexity**			**.0056**			.1903
1a	236 (79.2%)	272 (71.4%)		331 (76.4%)	177 (71.9%)	
1b	31 (10.4%)	76 (19.9%)		59 (13.6%)	48 (19.5%)	
1c	25 (8.4%)	23 (6.0%)		31 (7.2%)	17 (6.9%)	
2–3	6 (2.0%)	10 (2.6%)		12 (2.8%)	4 (2.8%)	
**Facility region**			**<.0001**			**<.0001**
Northeast	37 (12.4%)	59 (15.5%)		57 (13.2%)	39 (15.9%)	
Midwest	29 (9.7%)	49 (12.9%)		45 (10.4%)	33 (13.4%)	
South	90 (30.2%)	133 (34.9%)		135 (31.2%)	88 (35.8%)	
West	19 (6.4.%)	88 (23.1%)		49 (11.3.%)	58 (23.6%)	
Other	123 (41.3%)	52 (13.6%)		147 (33.9%)	28 (11.4%)	
*Median days (IQR)*						
**Length of stay**	11.0 (14.0)	30.0 (65.0)	**<.0001**	12.0 (17.0)	40.0 (80.0)	**<.0001**
**Admission to culture**	0.0 (1.0)	3.0 (20.0)	**<.0001**	0.0 (2.0)	5.0 (28.0)	**<.0001**
**Culture to contact Precautions**	0.0 (2.0)	9.0 (19.0)	**<.0001**	2.0 (4.0)	22.0 (40.0)	**<.0001**
**Culture to discharge**	9.0 (11.0)	19.0 (36.0)	**<.0001**	11.0 (12.0)	25.0 (48.0)	**<.0001**

Note. IQR, interquartile range. Bold indicates that the *P*-value is less than 0.05.

Most (63.8%) patients had evidence of CP within 7 days of the culture date (Table 1). After examination, factors associated with precautions within 7 days were very similar to those within 3 days. Again, those with evidence of precautions were older than those without (mean = 73.8 yr (11.7) vs 69.4 (11.4), *P*-value < .0001). Length of stay was again significantly shorter for those whose precautions occurred within 7 days compared with those without (median 12 d vs 40 d (*P*-value < .0001). The length of time from admission to culture was again significantly shorter for those with precautions within 7 days compared with those without (median 0 d vs 5 d (*P*-value < .0001).

## Discussion

In this retrospective chart review of hospitalized patients with positive CRE cultures, less than half of patients had CP enacted within 3 days of the culture, and only 63.8% of patients were placed in CP within 7 days of their culture date. A key result of our study was that patients who did not receive CP had a longer overall length of stay and were cultured later in their admission compared with those who did not receive CP. This finding is particularly interesting because length of stay is considered a primary risk factor for hospital-acquired CRE.^2,5^ This result was found independent of any confounding by indication as the entire cohort consisted of patients who were culture positive. These findings may indicate some decrease in adherence to CP guidelines in patients with longer admissions.

This study has several strengths. The use of a national cohort allowed for the evaluation of CP amid a wide geographic location as well as in hospitals of varying sizes. Furthermore, CRE is a rare infection, and this large cohort allowed for the analysis of CP for over 600 hospitalized patients with CRE infection. This work also has some limitations; the VA primarily consists of elderly White male patients; therefore, results from this work may not be generalizable to the US population. The nature of the data did not allow for a more nuisance analysis; instead, the 3- and 7-day time points were used. Although every effort was made to identify each instance of CP within this cohort, some may still have been missed. In conclusion, greater than 1 in 3 patients with CRE did not have evidence of initiation of CP even by 7 days after their culture date, a time at which nearly all cultures should have been reported as CRE or presumptive CRE by the microbiology laboratory. Given the critical role CP plays in combating hospital CRE transmission, additional research is needed to understand barriers to CP initiation.
